# Malformation of the Heart in a Child

**Published:** 1844-09-21

**Authors:** C. D. Meigs

**Affiliations:** Professor of Obstetrics in Jefferson Medical College, Philadelphia


### THE MEDICAL EXAMINER,

### And Record of Medical Science.

Vol. VII.] PHILADELPHIA, SATURDAY, SEPTEMBER 21, 1844. [No. 19.

### MALFORMATION OF THE HEART IN A CHILD,

By C. D. Meigs, M. D., Professor of Obstetrics in Jefferson Medical College, Philadelphia.

To the Editor of the Medical Examiner.

Sir,—At 7 P. M. of the 11th inst. I was called to Mrs. —— a young lady, in labour with her first child. Pains had been occasionally felt since 8, A. M., but were now frequent and sharp. The vertex presented in the 1st position; the os uteri was half an inch in diameter and extremely thin; the membranes not ruptured. A little before 10 the waters came off, and the os uteri yielded; the pains were very regular and strong, with perfect intervals betwixt each pain. At 11 o'clock the head was born, and fell as if the neck were relaxed, into my hand, but it soon stiffened; and the child immediately breathed; at the next effort, the shoulders came, and were followed by the body; as the trunk came away the child breathed again with a powerful inspiration. It was laid upon its back on the bed, and seemed to be completely still; upon making affusion of cold spirit on the breast it again breathed strongly; and not again until renewed affusions of cold brandy caused it to respire; in fine after eight or ten respirations it died.

Various attempts to resuscitate the infant proved unavailing. I cut the cord in order to let blood; but not a drop escaped from the vessels; indeed I am not conscious of having felt more than two pulsations in the cord, which was of a dark colour like the cord of a child dead and putrid in utero. I need not say that baths, frictions, inflations of the lungs, &c., were tried.

As the child was of very large size and well formed and developed, and had been born by a perfectly natural and spontaneous parturition, the question as to the cause of its death embarrassed me—seeing that I could assign no certain reason for its loss.

The epigastrium, the hypochondria, and the superior half of the umbilical region appeared to be swollen and hard, apparently from a distended liver occupying the interior, and that was the only unnatural appearance discoverable in the infant.

On the 11th, at 12 o'clock, Dr. Ellerslie Wallace made an autopsy for me, and he discovered that the liver occupied at the least two-thirds of the abdominal cavity, and was of a deep black colour.

The incisions all oozed a dark venous looking blood, and one that was made on the convex surface of the liver bled very much.

The thymus gland might have weighed about 2½ to 3 drams.

The heart was of a natural size except that the right auricle was very large.

The iter ad ventriculum dextrum was large enough freely to admit of the little finger to pass through it; and the tricuspid valve could not close the iter on account of the immense size of the opening. The right anterior segment of the valve was well formed; the rest of it was so short and adhered so closely to the ventricular wall as to be wholly unfit for its office. The foramen Botalli was very large, and the valve behind it seemed insufficient to close it perfectly.

Having made this examination, I came to the following conclusion as to the causes of the child's death; which I submit for your judgement.

The tricuspid valve was nearly useless; hence the blood passing from the auricle into the right ventricle was mostly (not all) thrown back through the iter ad ventriculum and filled the auricle instead of flowing off to the ductus arteriosus and branches of the pulmonary artery. This regurgitation constantly retarded the blood in the cavæ; the blood in the hepatic veins returning from the umbilical vein and placenta was congested in the vessels of the liver so as to give to that viscus its enormous extension. The diaphragm was greatly embarrassed by the presence of the tumour of the liver.

When the child breathed upon its blood it forced very little into the pulmonary artery, nearly the whole mass being backed upon the venous trunks in the liver and elsewhere; particularly in the placenta, which was loaded with blood. The child died with *cyanosis*, from dilated iter ad ventriculum dextrum and imperfect action of its tricuspid valves, which faulty conformation led to the engorgement and tumefaction of the liver.

If you think proper to record this case and the explanation of it I have given, I presume it might be considered valuable, not only as accounting for a pathological state of the liver, and the sudden death of a child apparently well developed; but it might also be regarded as important in a medico-legal point of view, since the causes of death in still born children and neonati are the frequent subjects of medico-legal inquiry.

